# Draft Genome Sequence of *Pseudomonas chlororaphis* ATCC 9446, a Nonpathogenic Bacterium with Bioremediation and Industrial Potential

**DOI:** 10.1128/genomeA.00474-17

**Published:** 2017-06-08

**Authors:** Fabian Moreno-Avitia, Luis Lozano, Jose Utrilla, Francisco Bolívar, Adelfo Escalante

**Affiliations:** a Departamento de Ingeniería Celular y Biocátalisis, Instituto de Biotecnología, Universidad Nacional Autónoma de México (UNAM), Cuernavaca, Morelos, México; b Centro de Ciencias Genómicas, UNAM, Cuernavaca, Morelos, México

## Abstract

*Pseudomonas chlororaphis* strain ATCC 9446 is a biocontrol-related organism. We report here its draft genome sequence assembled into 35 contigs consisting of 6,783,030 bp. Genome annotation predicted a total of 6,200 genes, 6,128 coding sequences, 81 pseudogenes, 58 tRNAs, 4 noncoding RNAs (ncRNAs), and 41 frameshifted genes.

## GENOME ANNOUNCEMENT

Different strains of *Pseudomonas chlororaphis* have been reported as biocontrol agents over a wide variety of organisms (1–6). *P. chlororaphis* strain ATCC 9446 is a member of a genus which has features that are interesting for different applications. Its potential capability to degrade triphenyltin, an organotin compound known to cause harmful effects on a variety of aquatic organisms, has been studied (7, 8). Furthermore, this strain was reported to produce 2-keto-l-gulonic acid, l-carnitine, and 6-aminopenicillanic acid (9–11).

The genome of strain ATCC 9446v was sequenced at the Sequencing and Polymorphism Identification Unit of the Instituto Nacional de Medicina Genómica in a NextSeq 500 Illumina sequencer with a 2 × 150 paired-end indexed configuration, achieving a genome coverage of 120-fold. Assembly was performed with SPAdes version 3.9.0 and IDBA_UD version 1.1.1 parallel, and the contigs obtained were merged and optimized using METASSEMBLER version 1.5 (12–14). Contigs were reordered using MAUVE version snapshot_2015_02_13 using the genome of *P. chlororaphis* 189 as a reference (15).

The draft genome of *P. chlororaphis* strain ATCC 9446 consists of 6,783,030 bp assembled in 35 contigs, with an average G+C content of 63%; the contigs length average was 15,918 bp (*N50*, 843,135 bp). The draft genome was annotated using the NCBI Prokaryotic Genome Annotation Pipeline, which predicted a total of 6,200 genes, 6,128 coding sequences, 81 pseudogenes, 58 tRNAs, 4 noncoding RNAs (ncRNAs), and 41 frameshifted genes (16). Annotation revealed genes related to toluene tolerance, alkanoates, alginate, phenazines, nonribosomal peptides, and polyketide synthesis, among many other genes involved in diverse metabolic pathways with biotechnological applications.

### Accession number(s).

This whole-genome shotgun project was deposited at DDBJ/EMBL/GenBank under the accession number NBAT00000000. The version described in this report is BioProject PRJNA380114 and BioSample SAMN06627644.

## References

[1] Schmidt-Eisenlohr H, Baron C 2003 The competitiveness of *Pseudomonas chlororaphis* carrying pJP4 is reduced in the *Arabidopsis thaliana* rhizosphere. Appl Environ Microbiol 69:1827–1831. doi:10.1128/AEM.69.3.1827-1831.2003. 12620876 PMC150058

[2] Kupferschmied P, Maurhofer M, Keel C 2013 Promise for plant pest control: root-associated pseudomonads with insecticidal activities. Front Plant Sci 4:287. doi:10.3389/fpls.2013.00287. 23914197 PMC3728486

[3] Dimkpa CO, Zeng J, McLean JE, Britt DW, Zhan J, Anderson AJ 2012 Production of indole-3-acetic acid via the indole-3-acetamide pathway in the plant-beneficial bacterium *Pseudomonas chlororaphis* O6 is inhibited by ZnO nanoparticles but enhanced by CuO nanoparticles. Appl Environ Microbiol 78:1404–1410. doi:10.1128/AEM.07424-11. 22210218 PMC3294495

[4] Shen T, Liu Q, Xie X, Xu Q, Chen N 2012 Improved production of tryptophan in genetically engineered *Escherichia coli* with TktA and PpsA overexpression. J Biomed Biotechnol 2012:605219. doi:10.1155/2012/605219. 22791961 PMC3390157

[5] Loewen PC, Villenueva J, Fernando WGD, de Kievit T 2014 Genome sequence of *Pseudomonas chlororaphis* strain PA23. Genome Announc 2(4):e00689-14. doi:10.1128/genomeA.00689-14. 25035328 PMC4102865

[6] Wang GW, Hu WT, Huang BK, Qin LP 2011 *Illicium verum*: a review on its botany, traditional use, chemistry and pharmacology. J Ethnopharmacol 136:10–20. doi:10.1016/j.jep.2011.04.051. 21549817

[7] Inoue H, Takimura O, Fuse H, Murakami K, Kamimura K, Yamaoka Y 2000 Degradation of triphenyltin by a fluorescent pseudomonad. Appl Environ Microbiol 66:3492–3498. doi:10.1128/AEM.66.8.3492-3498.2000. 10919812 PMC92176

[8] Inoue H, Takimura O, Kawaguchi K, Nitoda T, Fuse H, Murakami K, Yamaoka Y 2003 Tin-carbon cleavage of organotin compounds by pyoverdine from *Pseudomonas chlororaphis*. Appl Environ Microbiol 69:878–883. doi:10.1128/AEM.69.2.878-883.2003. 12571007 PMC143631

[9] Huang HT, English AR 3 1966 Production of 6-aminopenicillanic acid. US patent 3239427 A.

[10] Sonoyama T, Kageyama B, Honjo T 11 1975 Process for producing 2-keto-l-gulonic acid. US patent 3922194 A.

[11] Yokozeki K, Kubota K 3 1987 Method of producing l-carnitine. US patent 4650759 A.

[12] Bankevich A, Nurk S, Antipov D, Gurevich AA, Dvorkin M, Kulikov AS, Lesin VM, Nikolenko SI, Pham S, Prjibelski AD, Pyshkin AV, Sirotkin AV, Vyahhi N, Tesler G, Alekseyev MA, Pevzner PA 2012 SPAdes: a new genome assembly algorithm and Its applications to single-cell sequencing. J Comput Biol 19:455–477. doi:10.1089/cmb.2012.0021. 22506599 PMC3342519

[13] Peng Y, Zhang MM, Chen ZF, Hu K, Liu YC, Chen X, Liang H 2013 Synthesis, characterization, and interaction with biomolecules of platinum(II) complexes with shikimic acid-based ligands. Bioinorg Chem Appl 2013:565032. doi:10.1155/2013/565032. 23533373 PMC3603162

[14] Wences AH, Schatz MC 2015 Metassembler: merging and optimizing de novo genome assemblies. Genome Biol 16:207. doi:10.1186/s13059-015-0764-4. 26403281 PMC4581417

[15] Darling ACE, Mau B, Blattner FR, Perna NT 2004 Mauve: multiple alignment of conserved genomic sequence with rearrangements. Genome Res 14:1394–1403. doi:10.1101/gr.2289704. 15231754 PMC442156

[16] Tatusova T, DiCuccio M, Badretdin A, Chetvernin V, Ciufo S, Li W 2013 Prokaryotic genome annotation pipeline. *In* the NCBI Handbook, 2nd ed National Center for Biotechnology Information, Bethesda, MD.

